# The role of Exo70 in vascular smooth muscle cell migration

**DOI:** 10.1186/s11658-016-0019-8

**Published:** 2016-09-22

**Authors:** Wenqing Ma, Yu Wang, Xiaomeng Yao, Zijian Xu, Liguo An, Miao Yin

**Affiliations:** 1 Key Laboratory of Animal Resistant Biology of Shandong, College of Life Science, Shandong Normal University, Jinan, 250014 People’s Republic of China; 2 Shandong Provincial Hospital affiliated to Shandong University, Jinan, 250014 People’s Republic of China; 3 No.10 High School of Zibo, Zibo, 255000 People’s Republic of China

**Keywords:** Exo70, Exocyst, Vascular smooth muscle cell, Cell migration, RNAi, Wound-healing assay, Immunohistochemistry, Transwell assay

## Abstract

**Background:**

As a key subunit of the exocyst complex, Exo70 has highly conserved sequence and is widely found in yeast, mammals, and plants. In yeast, Exo70 mediates the process of exocytosis and promotes anchoring and integration of vesicles with the plasma membrane. In mammalian cells, Exo70 is involved in maintaining cell morphology, cell migration, cell connection, mRNA splicing, and other physiological processes, as well as participating in exocytosis. However, Exo70’s function in mammalian cells has yet to be fully recognized. In this paper, the expression of Exo70 and its role in cell migration were studied in a rat vascular smooth muscle cell line A7r5.

**Methods:**

Immunofluorescent analysis the expression of Exo70, α-actin, and tubulin in A7r5 cells showed a co-localization of Exo70 and α-actin, we treated the cells with cytochalasin B to depolymerize α-actin, in order to further confirm the co-localization of Exo70 and α-actin. We analyzed Exo70 co-localization with actin at the edge of migrating cells by wound-healing assay to establish whether Exo70 might play a role in cell migration. Next, we analyzed the migration and invasion ability of A7r5 cells before and after RNAi silencing through the wound healing assay and transwell assay.

**Results:**

The mechanism of interaction between Exo70 and cytoskeleton can be clarified by the immunoprecipitation techniques and wound-healing assay. The results showed that Exo70 and α-actin were co-localized at the leading edge of migrating cells. The ability of A7r5 to undergo cell migration was decreased when Exo70 expression was silenced by RNAi. Reducing Exo70 expression in RNAi treated A7r5 cells significantly lowered the invasion and migration ability of these cells compared to the normal cells. These results indicate that Exo70 participates in the process of A7r5 cell migration.

**Conclusions:**

This research is importance for the study on the pathological process of vascular intimal hyperplasia, since it provides a new research direction for the treatment of cardiovascular diseases such as atherosclerosis and restenosis after balloon angioplasty.

## Background

The exocyst complex is composed of eight subunits such as Sec3, Sec5, Sec6, Sec8, Sec10, Sec15, Exo70 and Exo84 [1]. In yeast, the exocyst complex is mainly involved in the exocytosis process. Gene mutations in each component could result in accumulation of secretory vesicles and secretory dysfunction [2]. Different from the single function of other exocyst components, Exo70 participates in anchoring and fusing secretory and transport vesicles to the target plasma membrane, through interactions between small GTPase Rho family members [3]. Lastly, Exo70 regulates the exocyst complex assembly process [4–6].

In mammals, as an important component of the exocyst complex, Exo70 is involved in the exocytosis process of secretory vesicles, similar to its function in yeast [7, 8]. However, Exo70 exhibits diverse characteristics on location and function in mammalian cells. It is mainly located in the mammalian cell cytoplasm, cell junctions, and the peripheral area of the nucleus, where it is involved in the maintenance of cell morphology, cell migration, cell connections, mRNA precursorsplicing, and other physiological processes [8–11]. However, its function in mammalian cells has yet to be fully recognized.

Vascular smooth muscle cells (VSMC) are the mesenchymal cells of blood vessels. In the process of atherosclerosis (AS), VSMC change from contraction type to synthetic type, leading to increased proliferation, migration, and secretion capacities [12]. The migration of synthetic VSMC from tunica media to tunica intima leads to thickening of the intima. Since Exo70 is involved in the secretion and migration of other mammalian cells, we speculate that Exo70 should be expressed and play a role in the regulation of VSMC migration.

## Materials and methods

### Cell culture

Rat aortic smooth muscle cell line A7r5 was maintained at 37 °C in DMEM supplemented with 10 % FBS, 2 mmol/L l-glutamine, 100 U ml^−1^ penicillin, and 100 μg ml^−1^ streptomycin, in a 5 % CO_2_ incubator.

### RT-PCR

Fast Lane cell cDNA kit (Qiagen, 21651, Germany) was used for first strand cDNA synthesis directly from cultured cells. Housekeeping gene GAPDH was amplified by PCR using the cDNA synthesized as a template to verify the quality of the cDNA. Verified cDNA was used in Exo70 RT-PCR detection. The primer sequences used in this experiment were the following: for Exo70 gene, Exo70-f: 5’-CCCCAACAAGAGGAAAGA-3’ and Exo70-r: 5’-CCTGACAAAGGCACTAACG-3’; for GAPDH gene, GAPDH-f : 5’-AGAGACAGCCGCATCTTCTTG-3’ and GAPDH-r : 5’-GGTAACCAGGCGTCCGATAC-3’. Gel electrophoresis was performed to detect the PCR products. Exo70 PCR product size was 115 bp.

### Immunohistochemistry

A7r5 cells during their logarithmic phase, were dissolved to form a cell suspension and seeded in 24-well plates containing the coverslips in each well. When the cells reached 80 % confluence in the coverslips, the original culture was discarded, cells were washed with PBS, fixed with room-temperature 4 % paraformaldehyde (PFA)/PBS for 30 min, washed, permeabilized with 0.1 % Triton X-100/TBS for 30 min, and blocked with 0.5 % BSA. Next they were washed and sequentially incubated with primary antibody at 4 °C overnight and secondary antibody at room-temperature for 60 min. Cells were observed with a confocal microscope and recorded.

### RNAi protocol

Rat Exoc7 gene (NM_022691) was used as the target gene. According to the complete rat Exoc7 mRNA sequence in Gene Bank, four shRNA interference sequences were designed: pLV-ratExoc7-sh1 (GGAACCAAGATTTCATGAATG CTCGAG CATTCATGAAATCTTGGTTCC TTTTT), pLV-ratExoc7-sh2 (GGATAACATCAAGAATGATCC CTCGAG GGATCATTCTTGATGTTATCC TTTTT), pLV-ratExoc7-sh3 (GCCTAAAGATGGCACCGTTCA CTCGAG TGAACGGTGCCATCTTTAGGC TTTTT), and pLV-ratExoc7-sh4 (GCGCCATCTTCCTACACAACA CTCGAG TGTTGTGTAGGAAGATGGCGC TTTTT). These sequences were inserted into a lentivirus vector to construct four shRNA expression vectors and they were used to transfect A7r5 cells to verify the efficiencies of interference. The highest efficiency vector pLV-ratExoc7-sh2 was selected and the corresponding lentivirus was used for subsequent experiments. Cells were cultured for 12 h and then the culture medium was removed. Three ml DMEM high glucose complete medium with 10 % FBS without P/S was added, and the corresponding virus solution was added directly to the culture plate with MOI = 50. Twelve hours post-infection, the culture medium was changed with 5 ml DMEM high glucose complete medium with 10 % FBS. The cells were continuously cultured for 4 days with medium replacement every 2 days. Ninety-six hours post-infection, the infected cells were observed under the microscope. Fresh DMEM high glucose complete medium with 10 % FBS was added and 1 μg/ml of puromycin was added for selection. The cells were cultured for additional 4 days with daily medium change. After 4 days’ selection, the cells were basically stabilized. When the resistant cells were 90 % confluent, they were digested and passed with at a 1:4 ratio and cultured with 1 μg/ml puromycin for 4 days. After continuous subcultyre and selection, cells were finally carrying the stable resistance gene and could be passed on. Cells were then digested and harvested and the interference efficiencies were analyzed using Western blot and qRT-PCR technology.

### Western blot

A7r5 and A7r5-ratExoc7-KD cells were lysed and scraped off the culture plates in protein sample buffer containing 4 % SDS, 20 % glycerol, and 25 mM Tris, pH 7.6. Proteins were extracted and quantified. The resulting cell lysates were subjected to SDS-PAGE and transferred to nitrocellulose membranes for Western blot analysis, using a goat monoclonal anti-rat antibody (1:1000, sc-135082, Santa Cruz). A secondary antibody was applied and proteins were visualized by ECL chemiluminescence reagent kit, stabilization of the membrane, and photographed using a gel imaging system.

### qRT-PCR

qRT-PCR analysis was performed using BioRad qReal-time PCR detection system and THUNDERBIRD SYBR qReal-timePCR kit. The GAPDH gene was used as an internal reference to ensure relatively accurate mRNA expression and to avoid system and random errors during sample processing. Based on qPCR primer design principles and the complete ratExoc7 mRNA sequence from Gene Bank, qPCR primers were designed. The sequences were the following: for ratExoc7 gene: ratExoc7-f: 5’-AGCGACCAGCTCACTAAGAA-3’, RatExoc7-r: 5’-CACAGGGATGATGGAGTTCTCCA-3’, PCR product was 87 bp in length; for GAPDH gene: GAPDH-f: 5’-TGCACCACCAACTGCTTAGC-3’, GAPDH-r: 5’-GGCATGGACTGTGGTCATGAG-3’, PCR product was 87 bp in length. The melting curve was used for the analysis of the immune specificity of amplified products. The standard curve was used to compare the relative content of the target genes.

### Wound healing assay

A7r5 cells were seeded in 24-well plates (~1 × 10^4^ cells per well) for 24 h. When cells reached the confluence, the culture medium was removed, 2 ml fresh culture medium containing 10 μg ml^−1^ mitomycin were added, and the cells were cultured for additional 2 h. Next, scratches were introduced using a sterile 100 μl pipette tip. Cells were washed with DMEM to remove debris and the remaining cells were incubated with regular growth medium. Cells were observed after wounding at 0, 6, 12, 24 and 48 h by inverted microscope until the time point of complete wound closure.

### Transwell assay

A7r5 cells were cultured until 80 % confluence. The cells were digested with trypsin, washed with serum-free culture medium 3×, counted, and added to cell culture wells as cell suspensions with 1 × 10^4^ cells per well. Matrigel stored at −80 °C was transferred to a 4 °C refrigerator and was liquefied overnight. Sixty microliter Matrigel were then added into 300 μl serum-free culture medium and mixed. One hundred μl of this mixture was added to the above culture wells, and incubated at 37 °C for 4–5 h. The Matrigel was washed once with serum-free culture medium. One hundred μl cell suspensions were added to the wells. At the lower culture wells, 600 μl of 20 % FBS culture medium were added and the Transwell plate was incubated at 37 °C for 20–24 h. The Transwell was removed and washed with PBS twice, fixed with 5 % pentanediol at 4 °C. Crystal violet (0.5 %) was added for 5–10 min for staining. The wells were washed with PBS 2×, observed under the microscope, and cells were counted.

### Statistical analysis

Results are expressed as means ± SD. The comparisons between the two groups were made by one-way ANOVA using GraphPad PRISM 5.0. Values of *p < 0.05 were considered statistically significant.

## Results

### Exo70 expression and localization in A7r5 cells

**Fig. 1:**
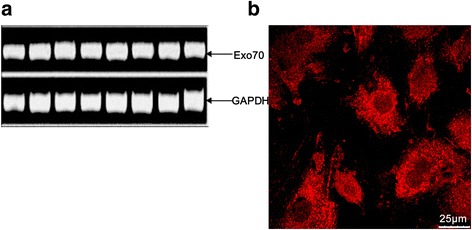
Exo70 expression and localization in A7r5 cells. **a** Exo70 expression in A7r5 cells evaluated by RT-PCR. **b** Exo70 immunofluorescent detection (*red*) in A7r5 cells. Exo70 marked by Cy3 is mainly distributed in the cytoplasm, but a small amount is also expressed in the nucleus. Scale length is 25 μm

The expression of the Exo70 gene in A7r5 cells was detected by RT-PCR and immunofluorescent staining. The results showed that Exo70 was expressed in A7r5 cells. Additionally, Exo70 expression was mainly localized in the cytoplasm and a small amount in the nucleus (Fig. 1a, b).

### Relationship between Exo70 expression and cytoskeleton localization in A7r5 cells

**Fig. 2:**
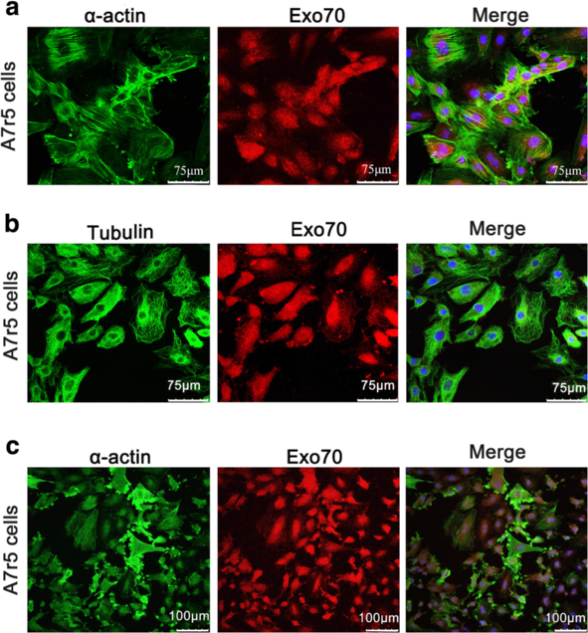
Interaction between Exo70 and the cytoskeleton in A7r5 cells. **a** α-actin and Exo70 co-localization in A7r5 cells. Immunofluorescent detection of α-actin (*green fluorescent*) and Exo70 (*red fluorescence*) expressions. The *yellow area* is a α-actin and Exo70 merged visualization, indicating their co-localization. Scale length is 75 μm. **b** Tubulin and Exo70 co-localization in A7r5 cells. Immunofluorescent detection of tubulin (*green fluorescent*) and Exo70 (*red fluorescence*) expressions. The *blue fluorescence* is showing the nuclei stained with DAPI. The *yellow area* indicating tubulin and Exo70 expression overlap is not present, suggesting the absence of co-localization. Scale length is 75 μm. **c** α-actin and Exo70 co-localization in A7r5 cells after 1 h treatment with cytochalasin B. Immunofluorescent detection of α-actin (*green fluorescent*) and Exo70 (*red fluorescence*) in A7r5 cells, the *blue fluorescence* is showing the nuclei stained with DAPI. The image on the left shows that α-Actin, Exo70, and the nucleus overlap, suggesting that α-actin depolymerization has occurred. Scale length is 100 μm

Immunofluorescent analysis in A7r5 cells showed that Exo70 was mainly located in the cytoplasm. If Exo70 is involved in cell migration, its function may be associated with the cytoskeleton. Immunofluorescent analysis of Exo70, α-actin, and tubulin in A7r5 cells showed a co-localization of Exo70 and α-actin (Fig. 2a), while Exo70 and tubulin did not show co-localization (Fig. 2b). In order to further confirm the co-localization of Exo70 and α-actin, the cells were treated with cytochalasin B to depolymerize α-actin. As shown in Fig. 2c, the cell’s actin filament network structure disappeared under the treatment. The localization of Exo70 was disordered and diffused in the cells, further indicating the existence of the co-localization relationship between Exo70 and α-actin in A7r5 cells.

### Exo70 role in A7r5 cell migration

During cell migration, Exo70 can directly interacts with the Arp2/3 complex [7, 9, 13]. The Arp2/3 complex generates a branched actin network that “pushes” the plasma membrane at the leading edges for cell migration [14–17]. To establish whether Exo70 might play a role in cell migration we analyzed Exo70 co-localization with actin at the edge of migrating cells.

**Fig. 3:**
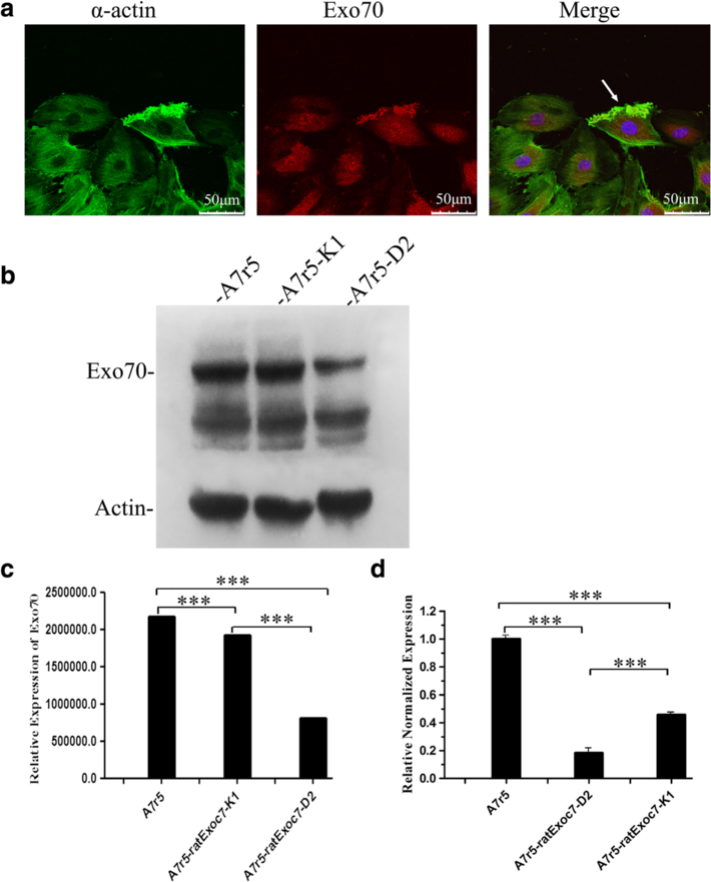
Exo70 location in the process of normal A7r5 cell migration. **a** A7r5 cells stably expressing GFP-tagged Exo70 were stained for α-actin (*green*), Exo70 (*red*) and nucleus (*blue*). The image on the right shows α-actin, Exo70, and the nucleus overlap. The yellow area indicates the overlap of α-actin and Exo70. The arrow points to α-actin and Exo70 together in the migration wound edges. Scale length is 50 μm. **b** Exo70 expression in A7r5 rat Exoc7-K1 and A7r5 rat Exoc7-D2 cells examined by western blot. β-actin was used as a loading control. **c** Quantification of Exo70 expression. ***p < 0.001. **d** Exo70 expression in the above siRNA-silenced cells was examined by qPCR. Relative Exo70 expression was normalized to untreated A7r5-control cells. ***p < 0.001

Immunofluorescence staining was used to analyze the co-localization of Exo70 and α-actin during the wound healing process. Figure 3a showed that Exo70 was localized at the edge of migrating A7r5 cells, where α-actin was also localized. This was consistent with the results of a previous study and showed that Exo70 and actin were co-localized at the edge of migrating A7r5 cells, with a co-localization rate of 48 %.

Using RNAi to suppress the expression of Exo70, two stable cell lines A7r5 rat Exoc7-K1 and A7r5 rat Exoc7-D2 were obtained through screening. Western blot and qRT-PCR techniques were used to analyze the expression of Exo70 in normal and RNAi silenced A7r5 cells. The results showed that the Exo70 expression levels in silenced cells were lower than that in the normal cells (Fig. 3b–d).

**Fig. 4:**
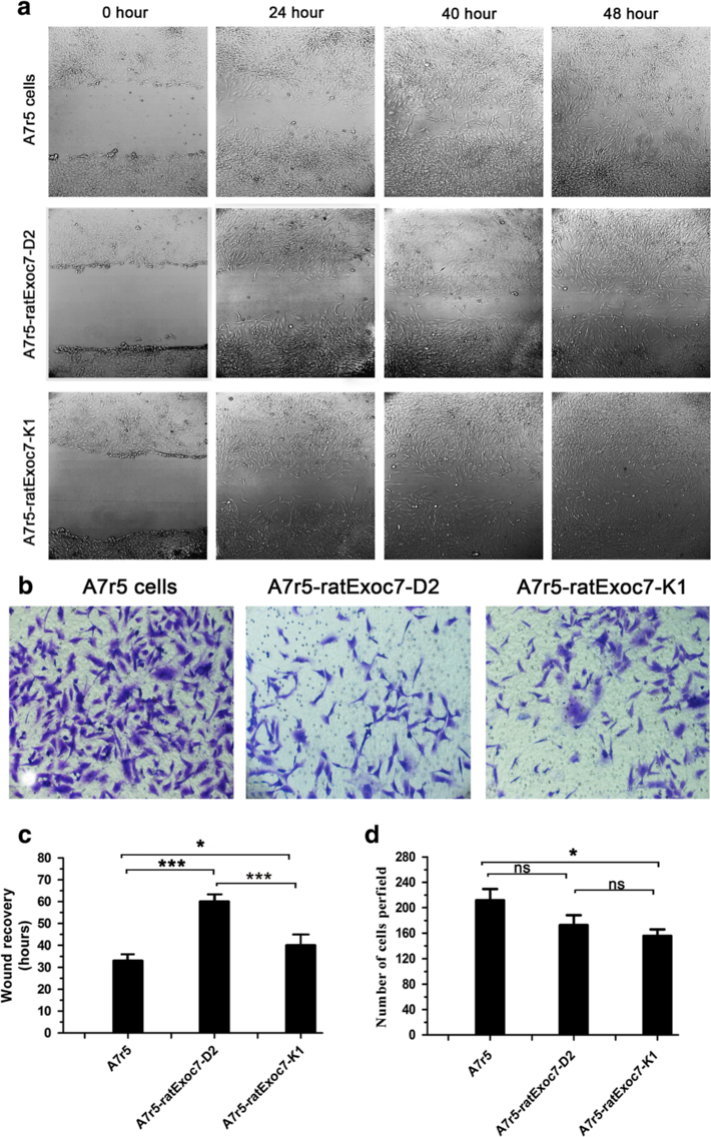
Exo70knockdown inhibits A7r5 cell migration and invasion. **a** Wound healing assay in different A7r5 cell lines. Images of the cells were taken at 0, 24, 40 and 48 h after the wound healing assay, magnification of 200 times. **b** Transwell assays of A7r5 cell migration. Images of the cells were taken and cells were counted 24 h after being seeded in the chambers. *n* = 3, three independent replicates. **c** Quantification of wound healing assay. Images of the A7r5 cells were taken every 8 h, and the time needed to reach a complete wound closure was determined. *n* = 3. Three independent clones of stable cell lines were tested. *p < 0.05; ***p < 0.001. **d** Quantification of migrating cells through transwell assay. The bars indicate the average number of migrating cells per field for each group. *n* = 10. *p < 0.05; ns, not statistically significant

Next, we analyzed the migration ability of A7r5 cells before and after RNAi silencing through the wound healing assay. Twenty-four hours after the introduction of the scratch, the scratch had healed, indicating the migration of the cells (Fig. 4a). Through data analysis, we found that scratch full healing time in normal A7r5 cells was 33.5 h, while RNAi silenced cells A7r5 rat Exoc7-K1 and A7r5 rat Exoc7-D2 were 42.1 and 61.2 h respectively (Fig. 4c). Therefore, when Exo70 was silenced, the cell migration ability was reduced accordingly.

### Exo70 role in A7r5 cell invasion

The Transwell assay was performed to detect cell invasion ability before and after RNAi silencing. The results showed that the number of RNAi silenced cells that invaded the lower chamber was significantly lower than that of untreated cells (Fig. 4b, d). Therefore, the reduced expression of Exo70 was corresponding to reduced cell invasion ability. This result indicated that Exo70 participated in A7r5 cell invasion and it further demonstrated that Exo70 might be involved in VSMC migration from tunica media to tunica intima during AS formation.

## Discussion

In different cell types, Exo70 is located in different parts of the cell. In yeast, Exo70 is mainly localized at the activated vesicle fusion site where the plasma membrane mediates the fusion process of the vesicles [3, 18]. In mammalian cells, the localization of Exo70 is more complicated. In adrenal pheochromocytoma cell line PC12, Exo70 is mainly distributed in the peripheral area of the nucleus of undifferentiated PC12 cells. After cell differentiation, the localization of Exo70 is moved from the peripheral area of nucleus to the growth cone of the neurite outgrowth [19]; in human hepatoma HepG2 cells, Exo70 is located near the microtubule organizing center [1]; in dog kidney epithelial cells MDCK, Exo70 is positioned at the cell plasma membrane region [11]; in HeLa cells, Exo70 can shuttle between cytoplasm and nucleus, and participates in nuclear mRNA splicing [10]. A7r5 cells are a synthetic type of VSMC with dual properties of secretion and transportation. Since Exo70 participates in the Golgi vesicle transport process, we speculated that Exo70 could be expressed in A7r5 cells. Our research showed that Exo70 was expressed in A7r5 cells and primarily localized in the cytoplasm. This result shows that Exo70 may participate in the secretion and migration processes of A7r5 cells.

The Arp2/3 complex is the main molecular regulator of actin polymerization. Activation of this complex is necessary for actin nucleation and the initiation of actin assembly [20]. Some researchers have found that Exo70 and the Arp2/3 complex are co-localized and Exo70 can directly interact with ARPC1, a subunit of the Arp2/3 complex [7, 21]. Two studies by Liu and one study by Zuo demonstrated that Exo70 directly interacts with the Arp2/3 complex [9], promotes actin filament nucleation and branching [22], and the Exo70 staining co-localized with actin puncta at sites of matrix degradation [7], indicating that Exo70 and actin are co-localized. Additional experiments will be included in our further research to obtain a more complete and clear insight of the interaction between Exo70 and Arp2/3 complex. In addition, studies by S. Wang and S.C. Hsu have found that Exo70 and the spindle structure are co-localized in PC12 cells, and are co-immunoprecipitated and co-purified with microtubules [19]. Further studies have shown that rat kidney cells NRK, over expressing Exo70, leads to the disintegration of the microtubule network; in HepG2 cells, Exo70 exists near the microtubule organizing centers, promoting secretory vesicle transport [23]; in the fruit fly*D. Melanogaster* and lipid cells, Exo70 reduced expression correspond to a reduced number of secretory vesicles at the plasma membrane, with Exo70 and microtubules showing the usual co-localization [24]. All these studies have shown that Exo70 function in different cells is related to its location. In this study, using an immunofluorescence technique, we specifically labeled Exo70, α-actin, and tubulin in A7r5 cells, and observed their localization under a confocal microscope. Our experimental results performed on A7r5 cells showed that Exo70 was mainly located in the cytoplasm and was co-localized with α-actin. We speculated that Exo70 may participate in vesicle transportation, secretion, and migration processes in A7r5 cells through its interaction with microfilaments. Our present work represents a preliminary research on the relationship between Exo70 and cytoskeleton localization in A7r5 cells. FRET and immunoprecipitation techniques can clarify in a greater extent the mechanism of interaction between Exo70 and cytoskeleton. Thus these additional experiments will be included in our further research.

In the process of AS, under the influence of various stimulating factors, VSMC displays abnormal phenomena such as phenotype transformation and uncontrolled proliferation [25], changing from a normal contractile phenotype to a synthetic type, possessing migration and secretion characteristics [26, 27]. Cell migration is mainly due to the formation of actin branching at the edge of the plasma membrane resulting in membrane expansion [28, 29]. The study by Wei Guo also showed that on the edge of migrating cells, the interaction of Exo70 and the Arp2/3 complex promoted actin assembly [12], thus contributing to a leading-edge plasma membrane expansion and promoting cell migration and invasion [14–17]. Furthermore, a study by Irving E. Vega showed that, in rat renal NRK cells, Exo70 could be observed on the edge of migrating cells [19]; in HeLa cells, Exo70 expression inhibition could reduce the rate of cell migration [9]; in prostate cancer cells, reduction of the expression of Exo70 inhibited tumor cell migration and invasion [30]; in breast cancer cells, over expression of Exo70 promoted cell migration and invasion, and RNAi knocking-down its expression level inhibited cell migration and invasion [7].

In this study, our results showed that Exo70 was localized at the edge of migrating A7r5 cells, where α-actin also accumulated. This result suggests that Exo70 regulates A7r5 cell migration through participation in the construction of the actin filament network. Reducing Exo70 expression in RNAi treated A7r5 cells significantly lowered the invasion and migration ability of these cells compared to the normal cells, indicating that Exo70 indeed promotes A7r5 cell invasion and migration. Exo70 was mainly located in the cytoplasm of A7r5 cells and regulated the cell migration processes through interaction with microfilaments.

## Conclusion

We show that the Exo70 take an important role in vascular smooth muscle cell migration, the Exo70 interaction with α-actin promotes A7r5 cell invasion and migration. This finding is of utmost importance for the study on the pathological process of vascular intimal hyperplasia, since it provides a new research direction for the treatment of cardiovascular diseases such as atherosclerosis and restenosis after balloon angioplasty.

## Abbreviations

AS: Atherosclerosis
FRET: Fluorescence Resonance Energy Transfer
GAPDH: Glyceraldehyde-3-phosphate dehydrogenase
RT-PCR: Realtime-PCR
VSMC: Vascular smooth muscle cells

## References

[1] He B, Guo W (2009). The exocyst complex in polarized exocytosis. Curr Opin Cell Biol.

[2] Hsu SC, TerBush D, Abraham M (2004). The Exocyst Complex in Polarized Exocytosis. Int Rev Cytol.

[3] Adamo JE, Moskow JJ, Gladfelter AS, Viterbo D, Lew DJ, Brennwald PJ (2001). Yeast Cdc42 functions at a late step in exocytosis, specifically during polarized growth of the emerging bud. J Cell Biol.

[4] Moore BA, Robinson HH, Xu Z (2007). The crystal structure of mouse Exo70 reveals unique features of the mammalian exocyst. J Mol Biol.

[5] Jiu Y, Jin C, Liu Y (2012). Exocyst Subunits Exo70 and Exo84 Cooperate with Small GTPases to Regulate Behavior and Endocytic Trafficking in C. elegans. PLoS One.

[6] Ren J, Guo W (2012). ERK1/2 Regulate Exocytosis through Direct Phosphorylation of the Exocyst Component Exo70. Dev Cell.

[7] Liu J, Yue P, Artym VV, Mueller SC, Guo W (2009). The role of the exocyst in matrix metalloproteinase secretion and actin dynamics during tumor cell invadopodia formation. Mol Biol Cell.

[8] Thapa N, Sun Y, Schramp M, Choi S, Ling K, Anderson RA (2012). Phosphoinositide signaling regulates the exocyst complex and polarized integrin trafficking in directionally migrating cells. Dev Cell.

[9] Zuo X, Zhang J, Zhang Y, Hsu SC, Zhou D, Guo W (2006). Exo70 interacts with the Arp2/3 complex and regulates cell migration. Nat Cell Biol.

[10] Dellago H, Loscher M, Ajuh P, Ryder U, Kaisermayer C, Grillari-Voglauer R, Fortschegger K, Gross S, Gstraunthaler A, Borth N (2011). Exo70, a subunit of the exocyst complex, interacts with SNEV (hPrp19/hPso4) and is involved in pre-mRNA splicing. Biochem J.

[11] Xiong X, Xu Q, Huang Y, Singh RD, Anderson R, Leof E, Hu J, Ling K (2012). An association between type Igamma PI4P 5-kinase and Exo70 directs E-cadherin clustering and epithelial polarization. Mol Biol Cell.

[12] Lu H, Liu J, Liu S, Zeng J, Ding D, Carstens RP, Cong Y, Xu X, Guo W (2013). Exo70 isoform switching upon epithelial-mesenchymal transition mediates cancer cell invasion. Dev Cell.

[13] Liu J, Guo W (2012). The exocyst complex in exocytosis and cell migration. Protoplasma.

[14] Pollard TD, Boristy GG (2003). Cellular motility driven by assembly and disassembly of actin filaments. J Cell Biol.

[15] Goley ED, Welch MD (2006). The ARP2/3 complex: an actin nucleator comes of age. Nat Rev Mol Cell Biol.

[16] Insall RH, Machesky LM (2009). Actin dynamics at the leading edge: from simple machinery to complex networks. Dev Cell.

[17] Ridley AJ (2011). Life at the leading edge. Cell.

[18] Ponnambalam S, Baldwin SA (2003). Constitutive protein secretion from the trans-Golgi network to the plasma membrane. Mol Membr Biol.

[19] Vega IE, Hsu SC (2001). The Exocyst Complex Associates with Microtubules to Mediate Vesicle Targeting and Neurite Outgrowth. J Neurosci.

[20] Miller KG (2002). Extending the Arp2/3 complex and its regulation beyond the leading edge. J Cell Biol.

[21] Zhao Y, Liu J, Yang C, Capraro BR, Baumgart T, Bradley RP, Ramakrishnan N, Xu X, Radhakrishnan R, Svitkina T, Guo W (2013). Exo70 generates membrane curvature for morphogenesis and cell migration. Dev Cell.

[22] Liu J, Zhao Y, Sun Y, He B, Yang C, Svitkina T, Goldman YE, Guo W (2012). Exo70 stimulates the Arp2/3 complex for lamellipodia formation and directional cell migration. Curr Biol.

[23] Beronja S, Laprise P, Papoulas O, Pellikka M, Sisson J, Tepass U (2005). Essential function of Drosophila Sec6 in apical exocytosis of epithelial photoreceptor cells. J Cell Biol.

[24] Wang S, Hsu SC (2006). The molecular mechanisms of the mammalian exocyst complex in exocytosis. Biochem Soc Trans.

[25] Orr AW, Hastings NE, Blackman BR, Wamhoff BR (2010). Complex regulation and function of the inflammatory smooth muscle cell phenotype in atherosclerosis. J Vasc Res.

[26] Orlandi A, Bennett M (2010). Progenitor cell-derived smooth muscle cells in vascular disease. Biochem Pharmacol.

[27] Davis-Dusenbery BN, Wu C, Hata A (2011). Micro-managing Vascular Smooth Muscle Cell Differentiation and Phenotypic Modulation. Arterioscler Thromb Vasc Biol.

[28] Brymora A, Valova VA, Larsen MR, Roufogalis BD, Robinson PJ (2001). The brain exocyst complex interacts with RalA in a GTP-dependent manner: identification of a novel mammalian Sec3 gene and a second Sec15 gene. J Biol Chem.

[29] Dong G, Hutagalung AH, Fu C, Novick P, Reinisch KM (2005). The structures of exocyst subunit Exo70p and the Exo84p C-terminal domains reveal a common motif. Nat Struct Mol Biol.

[30] Cheung AY, Wu HM (2004). Overexpression of an Arabidopsis formin stimulates supernumerary actin cable formation from pollen tube cell membrane. Plant Cell.

